# Supplementary material for: WordCommentsAnalyzer: A windows software tool for qualitative research
Source: F1000Res. 2018 Sep 4;7:536. Originally published 2018 May 3. [Version 2] doi: 10.12688/f1000research.14819.2 (PMC6137411; doi:10.12688/f1000research.14819.2)
Supplement: Supplementary file 3 [file f1000research-7-17614-s0002.tgz › b2a79496-4ada-47c4-9682-a99ce867836d.zip!/Eight Iranian Health Journals/Yafteh _50.docx]

© Yafteh  . This work is licensed under CC BY 4.0 (https://creativecommons.org/licenses/by/4.0/).

Volume & Issue: دوره 8 - شماره 3

Title: تأثیر مکمل یاری آهن و روی به تنهایی و همراه با هم بر رتینول پلاسما در کودکان دبستانی

http://yafte.lums.ac.ir/article-1-1019-fa.html

مقدمه: کمبود ریزمغذیها یکی از مهمترین مشکلات بهداشتی جهان است. در بین ریزمغذیها کمبود آهن و روی شیوع بیشتری داشته و تداخل این دو ماده معدنی با ویتامین آ از اهمیت زیادی برخوردار است. هدف ازاین مطالعه مقایسه تأثیر مکمل یاری آهن و روی به تنهایی و همراه باهم بر وضعیت ویتامین آ در کودکان ابتدایی است. مواد و روش ها: در این مطالعه که بصورت کارآزمایی بالینی دو سوکور انجام گرفت، 81 دانش آموزپایه پنجم ابتدائی شهر خرم آباد به سه گروه تقسیم شدند. گروه اول مکمل آهن(20 میلی گرم آهن در روز)، گروه دوم مکمل روی(20 میلی گرم روی در روز) و گروه سوم مکمل آهن+ روی(20 میلی گرم آهن+20 میلی گرم روی) را به مدت 4 ماه دریافت نمودند. در آغاز و پایان مداخله رتینول پلاسما با روش HPLC اندازه گیری شد. داده ها با استفاده از برنامه نرم افزاری SPSS نسخه 5/11 تجزیه و تحلیل شدند. از آزمونهای ANOVA برای مقایسه میانگین بین گروهها و تی تست دوتایی برای مقایسه قبل و بعد از مداخله استفاده شد. در مورد فریتین پلاسما با توجه به اینکه توزیع غیر نرمال بود، از آزمون کروسکال والیس برای مقایسه استفاده شد.نتیجه گیری: رتینول پلاسما در هر سه گروه کاهش یافت. با توجه به اهمیت و نقش مهمی که رتینول در بدن بر عهده دارد پیشنهاد می شود در برنامه های مکمل یاری وضعیت ویتامین آ نیز در نظر گرفته شود. یافته ها: تغییرات رتینول پلاسما در گروههای دریافت کننده آهن به تنهایی، روی به تنهایی و توأم آنها به ترتیب 2/0±1/0-،1/0±1/0-،1/0±1/0- میکرومول بر لیتر بود. این تغییرات به تفکیک جنس و به ترتیب دختر و پسر در گروه آهن2/0±18/0- و 1/0±03/0-، روی 1/0±1/0- و 09/0±08/0-، آهن+روی 2/0±14/0- و 1/0±06/0- میکرومول بر لیتر بود. تفکیک گروهها بر اساس کمبود یا کفایت روی اولیه تأثیری بر نتایج نداشت.

Title: مقایسه اثر دیازپام و میدازولام بر روی توهم بعد از عمل ناشی از کتامین

http://yafte.lums.ac.ir/article-1-1020-fa.html

مقدمه: یکی از داروهایی که در بیهوشی عمومی کاربرد فراوان دارد، کتامین است. حلالیت دارو و ماهیت آن، قدرت بسیار بالای دارو برای القاء بیهوشی و عوارض اندک قلبی، عروقی و تنفسی آنرا داروی بسیار مناسبی برای بیهوشی ساخته است. اما این دارو عوارض جانبی از قبیل افزایش ترشحات راه هوایی، بالا رفتن فشار داخل مغزی، فشار داخل چشم، فشار داخل معده و… دارد که به سهولت قابل کنترل می باشد. مهمترین و شایعترین عارضه جانبی آن توهم بعد از عمل می باشد که شیوعی بین 5 تا 30% داشته و درمانی هم ندارد اما برای پیشگیری از آن عمدتاً بر استفاده از بنزودیازپین ها تأکید می شود. در این مطالعه اثر دیازپام و میدازولام را به عنوان پیش دارو در پیشگیری از توهم بعد از عمل ناشی از کتامین مورد بررسی قرار داده ایم. مواد و روش ها: در این کارآزمایی بالینی دوسوکور تعداد 50 نفر از بیمارانی که نامزد بیهوشی عمومی توسط کتامین بودند، به صورت تصادفی به دو گروه تقسیم شدند. در یک گروه بعنوان پیش دارو از دیازپام و در گروه دیگر از میدازولام استفاده شد، سپس شیوع توهم بعد از عمل در هر دو گروه محاسبه گردید و با استفاده از آزمون کای دو تفاوت آماری بین دو گروه بررسی شد. نتیجه گیری: نتایج نشان می دهد که میدازولام در پیشگیری از بروز توهم بعد از عمل ناشی از کتامین نسبت به دیازپام مؤثرتر است. به عبارتی اگر چه توهم بعد از عمل ناشی از کتامین درمان خاصی ندارد اما می توان با تجویز بنزودیازپین ها و به ویژه میدازولام از بروز آن پیشگیری نمود. یافته ها: تجزیه و تحلیل داده ها نشان داد که در گروه نخست که دیازپام دریافت نمودند، 36 درصد دچار توهم شدند و در گروه دیگر که میدازولام دریافت کردند تنها 8 درصد دچار توهم بعد از عمل شدند. آزمون آماری مجذور کای تفاوت آماری معنی داری را بین دو گروه از نظر بروز توهم بعد از عمل نشان داد.

Title: تأثیر مصرف خوراکی کپسول‌های حاوی غضروف کوسه ماهی بر پاسخ ایمنی سلولی بیماران مبتلا به سرطان پستان

http://yafte.lums.ac.ir/article-1-1021-fa.html

مقدمه: غضروف کوسه از جمله مکمل‌های دارویی مورد استفاده در درمان سرطان است که با مکانیسم‌های متعددی سبب مهار رشد تومور می‌شود. مدارک علمی کمی در خصوص نحوه تأثیر آن بر سیستم ایمنی انسان وجود دارد، لذا در این مطالعه اثر آن ‌را بر سیستم ایمنی سلولی بیماران مبتلا به سرطان پستان بررسی کردیم. مواد و روش ها: پس از تهیه پودرغضروف کوسه و توزیع آنها در کپسول‌های دارو، بسته‌های دارویی کدگذاری شدند. بیماران مبتلا به سرطان پستان علاوه بر هورمون درمانی، کپسول‌های غضروف کوسه (گروه تست) و کپسول‌های نشاسته (گروه کنترل) را طبق پروتکل درمانی مصرف کردند. قبل و بعد از دوره درمانی از آنان خونگیری شد. پس از جداسازی سلول‌های تک هسته‌ای خون محیطی گروه تست و کنترل و کشت آنها در آزمایشگاه، میزان IL-4 و IFNγ تولید شده توسط آنها به روش ELISA اندازه‌گیری گردید. درصد و نسبت سلول‌های T-CD4+ و T-CD8+‎ خون محیطی آنها با فلوسایتومتری قبل و بعد از درمان ارزیابی شد. بحث و نتیجه گیری: غضروف کوسه ماهی می‌تواند با تقویت سیستم ایمنی سلولی که اهمیت فوق‌العاده‌ای در دفاع ضد توموری در بیماران مبتلا به سرطان پستان دارد، به عنوان مکمل درمانی مناسبی در کنار درمان‌های رایج این بیماران عمل کند. یافته ها: مصرف غضروف کوسه سبب افزایش ترشح IFNγ و افزایش نسبت سلول‌های T-CD8+ به T-CD4+در بیماران گروه تست در مقایسه با قبل از درمان شد. در حالی که تفاوت معنی‌داری در این پارامترها در گروه کنترل دیده نشد.

Title: جا اندازی بسته و پین گذاری از راه پوست در درمان شکستگی های متافیز دیستال رادیوس

http://yafte.lums.ac.ir/article-1-1022-fa.html

مقدمه: شکستگی های متافیزیال دیستال رادیوس از شایعترین شکستگیهای سیستم اسکلتی بوده و شیوع آن به علت افزایش شیوع استئوپروز در حال افزایش است. هدف از مطالعه حاضر بررسی روش جااندازی و پین گذاری بسته در درمان این نوع شکستگی است. بحث و نتیجه گیری: با در نظر گرفتن سادگی روش جراحی و تهاجم بافتی کم و عوارض کمتر نسبت به دیگر روشها، جااندازی بسته و پین گذاری روش درمانی مناسبی جهت استفاده در شکستگی های متافیزیال دیستال رادیوس بخصوص با خردشدگی دورسال میباشد. یافته ها: جااندازی خوب و قابل قبول از لحاظ پرتونگاری در68 درصد موارد و نتایج عالی و خوب از لحاظ بالینی طبق معیار دمریت در 84 درصد موارد بدست آمد. در هیچ موردی تغییر شکل آشکاری در اندام بعد از درمان بوجود نیامد و هیچ یک از عوارض بوجود آمده موجب تأثیر عمده ای در کارآیی کلی فرد نشدند. مواد و روش ها: در این مطالعه تعداد 44 بیمارکه طبق تقسیم بندی یونیورسال در گروه 2 و در تقسیم بندی اولدر 2 و 3 بودند، در طی 3 سال مورد مطالعه قرار گرفتند. شکستگی بیماران تحت بیهوشی عمومی و با استفاده از فلوروسکوپی جااندازی بسته شده و توسط دو عدد پین فیکس شدند. نتایج درمان از لحاظ پرتونگاری با بررسی طول رادیال، زاویه رادیال و چرخش ولار و از لحاظ بالینی توسط معیار مدیفیه دمریت بررسی شد.

Title: بررسی ویژگی های فرهنگی، اقتصادی، اجتماعی و علل وابسته در بیماران

http://yafte.lums.ac.ir/article-1-1023-fa.html

مقدمه: سایکوز بعد از زایمان یک اختلال خلقی است که بطور متوسط 3-2 هفته بعد از زایمان شروع می شود. در موارد شدید علائم به حدی است که بیمار نمی خواهد از بچه اش مراقبت نماید و یا قصد صدمه رساندن جدی به بچه یا خودش یا هر دو را دارد. به نظر می رسد عوامل روانی اجتماعی متعددی در بروز این اختلال مؤثر باشد. پژوهش حاضر با هدف بررسی ویژگی فرهنگی، اقتصادی، اجتماعی و علل وابسته در بیماران مبتلا به سایکوز بعد از زایمان در سالهای 84-82 انجام گرفته است. مواد و روش ها: مطالعه حاضر یک مطالعه توصیفی – مقطعی است. جامعه مورد مطالعه شامل کلیه زنانی است که به فاصله 3 الی 12 هفته اول بعد از زایمان با یکی از علائم اختلال خلقی به درمانگاه تخصصی آموزشی – درمانی اعصاب و روان و مطب های خصوصی سطح شهر مراجعه نموده اند. نمونه گیری به روش سرشماری انجام گرفت. کلیه زنانی که 12-3 هفته از زایمان آنها گذشته و با یکی از علائم خلقی به درمانگاه تخصصی اعصاب و روان و مطب های خصوصی سطح شهر مراجعه نمودند، توسط متخصص روانپزشکی مورد معاینه و مصاحبه بالینی قرار گرفته و افرادی که مبتلا به اختلال افسردگی بعد از زایمان با شدت شدید و تابلوی سایکوتیک بودند، به عنوان نمونه در پژوهش وارد و مورد بررسی قرار گرفتند. اطلاعات پس از جمع آوری از طریق نرم افزار SPSS مورد تجزیه و تحلیل قرار گرفت. جهت توصیف فراوانی داده ها از آمار توصیفی استفاده گردید. بحث و نتیجه گیری: به منظور پیشگیری از بروز سایکوز بعد از زایمان شرایط مساعد جهت بارداری مادر فراهم گردد و با برنامه ریزی مناسب آموزشی زمینه حضور فرزند و تولد نوزاد در خانواده مهیا گردد. یافته ها: نتایج تحقیق نشان داد که اکثریت واحدهای مورد پژوهش در رده سنی 30-20 سال قرار داشته و خانه دار بودند. در اکثر موارد جنسیت نوزاد دختر و زایمانها به روش طبیعی انجام گرفته بودند. اکثر نمونه ها در روابط زناشویی خود مشکل داشته و دراکثر موارد حاملگی ها ناخواسته بوده است. همچنین یافته های تحقیق نشان داد که از بین 6/29% نمونه هایی که سابقه قبلی بیماری روانی داشته اند 1/9% سابقه افسردگی اساسی، 6/5% سابقه کج خلقی را ذکر نمودند. 5/18% از واحدهای مورد پژوهش سابقه بیماریهای جسمی نظیر تشنج، عفونت دستگاه ادراری، هیپرتیروئیدی، تشنج و کیست تخمدان را ذکر نمودند. یافته های تحقیق ارتباط معنی داری بین وجود سایکوز بعد از زایمان با متغیرهایی مثل سن، سطح تحصیلات، شغل، سابقه قبلی بیماری روانی، رتبه زایمان، ناهماهنگی در روابط زناشویی و تمایل به بچه دار شدن نشان داد.

Title: بررسی میزان رعایت اصول کلی پیشگیری از عفونتهای بیمارستانی در بیمارستانهای آموزشی خرم آباد 84-1383

http://yafte.lums.ac.ir/article-1-1024-fa.html

مقدمه: عفونت بیمارستانی یکی از مهمترین مشکلات بهداشتی درمانی محسوب می شوند. 10-7 درصد بیماران بستری در بیمارستان، حداقل به یک عفونت بیمارستانی مبتلا می شوند. پژوهش حاضر به منظور تعیین میزان رعایت اصول پیشگیری از عفونتهای بیمارستانی در بیمارستانهای آموزشی شهر خرم آباد در سالهای 84-1383 انجام شد. بحث و نتیجه گیری: آموزش مداوم کارکنان در رده های مختلف شغلی، استفاده از وسایل و روش های کاهش عفونت، افزایش منابع و امکانات و ارتقاء فضای فیزیکی، تأسیس سیستم ملی نظارت و کنترل مستمر عفونتهای بیمارستانی، بررسی اپیدمیولوژیک سالانه، و انجام کشت منظم، راهکارهای مهمی برای کنترل عفونت هستند که باید در بیمارستانهای آموزشی اجرا شوند. یافته ها: نتایج نشان داد رعایت اصول کلی پیشگیری از عفونتهای بیمارستانی در اکثر کارکنان (7/53%) در حد متوسط و شستن دستها در 90% موارد در حد ضعیف بود. از نظر فضای فیزیکی 83/47% و در مورد منابع و امکانات 3/78% بخش ها جهت پیشگیری از عفونتهای بیمارستانی در حد متوسط بودند. مواد و روش ها: این پژوهش یک مطالعه توصیفی مقطعی است که روی کلیه بخشهای (23 بخش) بیمارستانهای آموزشی شهر خرم آباد (بجز اتاق عمل) انجام شد. فضای فیزیکی، منابع و امکانات و عملکرد کارکنان در خصوص کنترل عفونت مورد مشاهده و بررسی قرار گرفت. اطلاعات از طریق دو چک لیست جمع آوری شد. وضعیت بخش ها و عملکرد کارکنان براساس امتیاز کسب شده از چک لیست ها در سه سطح خوب، متوسط و ضعیف طبقه بندی شدند. اطلاعات جمع آوری شده با استفاده از نرم افزارSPSS نسخه 11 و آزمونهای آماری توصیفی و استنباطی تجزیه و تحلیل گردید.

Title: بررسی ریسک انتقال ویروس هپاتیت C بدنبال آندوسکوپی دستگاه گوارش

http://yafte.lums.ac.ir/article-1-1025-fa.html

مقدمه: عفونت هپاتیت (HCV) C یکی از مسائل بهداشتی مهم در کشور ما می باشد. تصور می شود که انتقال ویروس هپاتیت C از طریق دستگاههای آندوسکوپی بندرت اتفاق می افتد. هدف این مطالعه بررسی میزان انتقال هپاتیت C بدنبال آندوسکوپی بعد از ضد عفونی به روش رایج و معمولی می باشد. بحث و نتیجه گیری: به نظر نمی رسد که آندوسکوپی که به روشهای معمولی ضد عفونی می شود نقش عمده ای در انتقال عفونت هپاتیت C داشته باشد و روش ضد عفونی مناسب از انتقال آن جلوگیری می کند. یافته ها: در 5 بیمار (1%) نتایج آنتی بادی HCV مثبت گزارش شد. در 3 نفر از بیماران فوق عفونت HCV به روش PCR تأیید شده است. در گروه کنترل نیز 6 نفر آنتی بادی HCV مثبت داشتند(2/1%). انتقال خون، سابقه تزریق دارویی غیر بهداشتی، دستکاریهای دندانپزشکی از عوامل خطر مستقل عفونت هپاتیت C در گروه بیماران و کنترل بودند. تفاوت آماری قابل توجهی بین گروه بیماران و کنترل از نظر میزان شیوع آنتی بادی HCV دیده نشد (74/0=p). مواد و روشها: در طی یک مطالعه آینده نگر 456 مورد بیمار که جهت آنها آندوسکوپی فوقانی انجام شده است مورد بررسی قرار گرفته اند. دستگاههای آندوسکوپی به روش معمولی با گلوتارالدئید 2% و به مدت 5 دقیقه ضد عفونی شده اند. آنتی بادی HCV جهت تمام بیماران چک شده و جهت بررسی سایر ریسک فاکتورهای هپاتیت C نیز پرسشنامه اطلاعات فردی بیماران ثبت شده است. شش ماه و یکسال بعد مجدداً تست HCV آنتی بادی کنترل و نتایج مثبت به روش PCR ارزیابی شده اند. پانصد فرد دهنده سالم خون بعنوان گروه کنترل مورد مطالعه قرار گرفته اند.

Title: مقایسه تأثیر یک ترکیب گیاهی با محلول مینوکسیدیل 2% در درمان تلوژن افلوویوم

http://yafte.lums.ac.ir/article-1-1026-fa.html

مقدمه: تلوژن افلوویوم یک بیماری خود محدود شونده غیر اسکار گذار است که خود را بصورت ریزش منتشر موهای سر نشان می دهد و معمولاً 3 ماه بعد از یک بیماری حاد اتفاق می افتد. از لحاظ تئوری محلول مینوکسیدیل بعنوان درمان این حالت پیشنهاد شده است. در این مطالعه، ما تأثیر محلول مینوکسیدیل و یک داروی گیاهی مشتمل بر گزنه دوپایه، رازیانه، دم اسب، آویشن و بابونه آلمانی را در درمان تلوژن افلوویوم مورد بررسی قرار دادیم. نتیجه گیری: بر طبق نتایج حاصل از این مطالعه، می توان نتیجه گیری نمود که داروی گیاهی در درمان تلوژن افلوویوم مؤثر است، هر چند که مطالعات بیشتر در این زمینه ضروری است. یافته ها: میانگین مدت زمان طول کشیدن تلوژن افلوویوم در بیماران استفاده کننده از مینوکسیدیل معادل 17 هفته و در گروه استفاده کننده از داروی گیاهی معادل 7 هفته بود. این اختلاف از لحاظ آماری معنی دار بود. مواد و روش ها: این مطالعه، یک مطالعه آینده نگر دوسوکور بود که در آن 24 بیمار مبتلا به تلوژن افلوویوم بصورت تصادفی به 2 گروه تقسیم شده و تحت درمان با مینوکسیدیل و داروی گیاهی قرار گرفتند. اطلاعات بدست آمده توسط آزمونهای آماری مورد تجزیه و تحلیل قرار گرفت.

Title: بررسی تأثیر عصاره های آبی والکلی گیاه آویشن شیرازی بر روی اشریشیا کلی

http://yafte.lums.ac.ir/article-1-1027-fa.html

مقدمه: به دلیل مقاومت روز افزون باکتری های بیماریزا به آنتی بیوتیک های جدید محققین در پی یافتن مواد ضد میکروبی با منشأ گیاهی به عنوان جایگزین آنتی بیوتیکهای غیر مؤثر هستند. هدف از انجام این مطالعه، بررسی خواص ضد باکتریایی عصاره آبی و الکلی گیاه آویشن شیرازی بر روی سویه های بالینی واستاندارد اشریشیا کلی انترو هموراژیک می باشد. مواد و روش ها: برگ های جوان گیاه پس ازخشک شدن به طور جداگانه به میزان 10گرم دردسی لیتربه اتانول 85 درجه وآب مقطر اضافه شده، سپس عصاره ها به روش تقطیر در خلاء استخراج شدند. خواص ضد باکتریایی عصاره ها ابتدا به روش رقت در لوله تعیین و سپس وزن خشک عصاره ها در هر میلی لیتر محاسبه و حداقل غلظت مهار کنندگی وکشندگی آنها بدست آمد. معادل این غلظت ها به چاهک های حفر شده در محیط مولر- هینتون آگار اضافه ومیانگین قطر هاله های عدم رشد برای عصاره ها و سویه ها مقایسه گردید. به منظورشناسایی مواد مؤثره ضد باکتریایی در عصاره های آبی و الکلی از روش کروماتوگرافی با صفحه نازک استفاده گردید. نتیجه گیری: عصاره الکلی آویشن شیرازی دارای اثرات چشمگیری بر روی سویه های اشریشیا کلی انترو هموراژیک می باشد ولی معرفی آن به عنوان یک ترکیب ضد باکتریایی نیاز به تحقیقات وسیع تری دارد. یافته ها: غلظت های معینی ازعصاره الکلی دارای خواص ضد باکتریایی قابل توجهی بود. در غلظت 78/0 میلی گرم در میلی لیتر دارای اثر مهاری و اثر کشندگی بر روی هر سه سویه بود و از این نظر سویه ها با هم تفاوتی نداشتند. همچنین تأثیر عصاره با کم شدن غلظت آن در چاهک ها کم می شد. این در حالی بود که عصاره آبی در هیچکدام ازغلظت ها بر روی سویه های استاندارد و بالینی مؤثر نبود.

Title: مقایسه روش های تغلیظ و خالص سازی کیست ژیاردیا

http://yafte.lums.ac.ir/article-1-1028-fa.html

مقدمه: ژیاردیا لامبلیا از شایع ترین تک یاخته های روده ای انسان در سراسر جهان می باشد. تشخیص آن عمدتاً از طریق مشاهده مستقیم صورت می گیرد ولی روش های مبتنی بر IFA و ELISA نیز کاربردهای تحقیقاتی و سرواپیدمیولوژی دارد. برای تهیه آنتی بادی های پلی کلونال به هدف طراحی کیت های ELISA و IFA، مطالعات مولکولی و ژنومی، آزمایشاتی نظیر PCR و کشت انگل و سایر تحقیقات اپیدمیولوژی،نیاز به خالص سازی کیست های انگلی می باشد. روش های مختلفی برای این منظور منتشر شده است و در این بین روشی که بتواند کیست ها را در طی روند خالص سازی بطور سالم و زنده نگه داشته و نیز تقریباً عاری از سایر آلودگی های مدفوعی و مواد اضافه باشد، نیاز می باشد. لذا در این مطالعه ما بر آن شدیم که نمونه های مثبت را با چندین روش متداول خالص سازی کرده و از نظر درجه خلوص با همدیگر مقایسه کنیم. مواد و روش ها: نمونه های مثبت طی سه روش جداگانه گرادیان یک مرحله ای سوکروز، گرادیان سوکروز-پرکول و گرادیان دو مرحله ای سوکروز در دو مرحله، مورد خالص سازی قرار گرفت. در نهایت نتایج حاصل از هر سه روش فوق از نظر درجه خلوص کیستی و میزان حذف مواد اضافی مدفوع و نیز میزان کیست های باز یافتی با لام مستقیم ولام نئوبار مورد بررسی قرار گرفت. یافته ها: محلول کیستی حاصل از روش اول و همچنین دوم حاوی کمی مواد دبری و سلولزی بود، در صورتی که در روش سوم، باکتری ها تقریبا حذف شده و کیست ها سالم بدست آمدند ولی میزان باز یافت آن کمتر بود(از 2گرم مدفوع حدود 104 ×3). نتیجه گیری: روش سوم (گرادیان دو مرحله ای سوکروز) در خالص سازی و ایزولاسیون کیست های انگل نسبت به دو روش قبلی مناسب تر بوده و میزان مواد اضافی مدفوع کاهش پیدا می کند ولی با اینحال ابداع روشی ساده که بتواند مواد اضافی و زاید را تا حد زیادی حذف نموده و از طرفی میزان بازیافتی کیست ها را بالا ببرد، ضروری به نظر می رسد.

Title: تأثیر تعداد وعده های غذایی بر پروفایل ایمونوگلوبولین ها و انسولین سرم در موش صحرایی

http://yafte.lums.ac.ir/article-1-1029-fa.html

مقدمه: گرچه مصرف سه و کمتر از سه وعده در روز ( رژیم کم وعده ) در بسیاری ازکشورها رایج میباشد ولی در حال حاضر در کشورهای غربی گرایش فزاینده ای به سوی تعداد وعده های غذایی بیشتر ولی با حجم کمتر(رژیم پر وعده) مشاهده میگردد. تا بحال ، اثرات تعداد وعده های غذایی بر ایمنی هومورال در حیوانات آزمایشگاهی مورد بررسی قرار نگرفته است. همچنین اطلاعات انسانی و حیوانی در مورد اثرات تعداد وعده های غذایی بر روی میزان انسولین سرم نیز ضد و نقیض است. هدف از مطالعه حاضر بررسی اثرات تعداد وعده های غذایی بر پروفایل ایمونوگلوبولین ها برای اولین بار روی موش صحرایی می باشد. همچنین در مطالعه اخیر، اثر تعداد وعده های غذایی روی میزان انسولین سرم نیز مورد بررسی قرار خواهد گرفت. مواد و روش ها: در یک مطالعه تجربی قبل و بعد مدل حیوانی، 30 موش صحرایی ماده از نوع Wistar با 11 هفته سن (15±210 گرم) بعد از 10 روز دوره سازگاری وزن و به طور تصادفی به دو گروه کاملاً مساوی تقسیم گردیدند.حیوانات غذای یکسان را یا بصورت 8 وعده به فواصل هر 2 ساعت یکبار از ساعت 6 بعد از ظهر (رژیم پر وعده) و یا بصورت 2 وعده در ساعات 9 شب و 6 صبح ( رژیم کم وعده ) به مدت 60 روز دریافت نمودند. از هر حیوان قبل و بعد از مداخله خون گرفته شد. جهت اندازه گیری میزان انسولین و ایمونوگلوبولین های سرم از ELISA استفاده گردید. تجزیه و تحلیل داده ها با روش آماری Student’s t-test صورت گرفت . پروژه تحقیقاتی توسط کمیته اخلاق پزشکی دانشگاه علوم پزشکی تبریز مورد مطالعه و به تصویب رسید. بحث و نتیجه گیری: رژیم غذایی پر وعده اثرات بهتری بر روی میزان انسولین، IgM و IgG سرم نسبت به رژیم کم وعده دارد .تحقیقات بیشتر در این زمینه روی انسان و حیوان توصیه میگردد. یافته ها: هر دو رژیم غذایی باعث کاهش غیر معنی داری (05/0> P ) در مقادیر انسولین و IgA سرم بعد از 60 روز مداخله گردیدند. گرچه در هر دو رژیم غذایی میزان انسولین سرم بطور غیر معنی دار کاهش پیدا کرده بود، اما درصد کاهش در رژیم پر وعده در قیاس با رژیم کم وعده بیشتر بود (3/5%- در برابر3/2%- ). میزان IgMو IgG سرم در هر دو رژیم غذایی بطور معنی داری(05/0< P ) افزایش پیدا کرده بود، اما اختلاف بین دو رژیم قبل و بعد از مداخله معنی دار (05/0p<) نبود. گرچه، در هر دو گروه میزان IgMو IgG سرم بطور معنی داری(05/0< P ) افزایش پیدا کرده بود، اما درصد افزایش در رژیم پر وعده در قیاس با رژیم کم وعده بیشتر بود(به ترتیب 56% در مقابل 38% و 37% در مقابل7/28% برای IgM و IgG سرم ). دریافت آب و غذا بطور غیر معنی داری(05/0> P ) در رژیم کم وعده در قیاس با رژیم پر وعده کمتر بود.

Title: بررسی اثر ویتامین D3 در ارتشاح لکوسیتی به مغز موشهای مبتلا به آنسفالومیلیت خود ایمن تجربی

http://yafte.lums.ac.ir/article-1-1030-fa.html

مقدمه: در بیماریهایی مثل مولتیپل اسکلروزیس و آنسفالومیلیت خودایمن تجربی ارتشاح لکوسیتی به داخل سیستم عصبی مرکزی در پاتوژنز و پیشرفت بیماری مؤثر می باشد. برخی بررسی ها نشان می دهد که تجویز ویتامین D3(فرم فعال ویتامین D) به موشهای مبتلا به EAE، شدت بیماری را تعدیل می کند. هدف از این مطالعه، بررسی کار آمدی ویتامین D3‎‏ ‏ در مهار EAE، اثر ویتامین D3‎‏ ‏ روی میزان ارتشاح لکوسیتی به مغز موشهای مبتلا به EAE مورد مطالعه می باشد. مواد و روش ها: موش های نر نژاد C57BL/6 در دو گروه درمانی (هر گروه 8 رأس) با شرایط سنی و وزنی مشابه قرار گرفتند. موش های گروه مطالعه (مبتلا به EAE تحت درمان با ویتامین D3) یک روز در میان 5 میکروگرم ویتامین D3‎‏ ‏ به ازای هر کیلوگرم وزن بدن به صورت داخل صفاقی از سه روز قبل تا 19 روز پس از ایجاد بیماری دریافت کردند. موش های گروه شاهد (مبتلا به EAE درمان نشده) تنها حلال ویتامین را با همان جدول زمانی دریافت نمودند. همچنین 5 رأس موش نر نژاد C57BL/6 با شرایط سنی و وزنی مشابه به عنوان گروه سالم در نظر گرفته شد. یافته ها: نتایج نشان داد که شدت علائم کلینیکی در موش های تحت درمان با ویتامین D3‎‏ ‏ (8/0±2/3) در مقایسه با گروه درمان نشده(44/0±3/5) بطور معنی داری کمتر می باشد (001/0p<). همچنین اختلاف معنی داری در تعداد سلولهای ارتشاح یافته به مغز، بین موشهای مبتلا به EAE درمان شده با ویتامین D3‎ و درمان نشده مشاهده شد (01/0p<). بحث و نتیجه گیری: این نتایج مشخص می نماید که درمان با ویتامین D3‎‏ ‏ موجب کاهش ارتشاح لکوسیت ها به مغز موشهای مبتلا به EAE شده و سبب کاهش ضایعات پاتولوژیک و علائم کلینیکی و تعدیل بیماری می گردد. لذا ویتامین D3‎‏ ‏ ممکن است در درمان بیماریهای التهابی همراه با ارتشاح سلولی به داخل بافت، مفید باشد.
